# Patients' experiences of, and engagement with, remote home monitoring services for COVID‐19 patients: A rapid mixed‐methods study

**DOI:** 10.1111/hex.13548

**Published:** 2022-07-07

**Authors:** Holly Walton, Cecilia Vindrola‐Padros, Nadia E. Crellin, Manbinder S. Sidhu, Lauren Herlitz, Ian Litchfield, Jo Ellins, Pei Li Ng, Efthalia Massou, Sonila M. Tomini, Naomi J. Fulop

**Affiliations:** ^1^ Department of Applied Health Research University College London London UK; ^2^ Department of Targeted Intervention University College London London UK; ^3^ Nuffield Trust London UK; ^4^ School of Social Policy, Health Services Management Centre, College of Social Sciences University of Birmingham Birmingham UK; ^5^ Institute of Applied Health Research, College of Medical and Dental Sciences University of Birmingham Birmingham UK; ^6^ Department of Public Health and Primary Care University of Cambridge Cambridge UK

**Keywords:** care, COVID‐19, patient engagement, patient experience, remote home monitoring

## Abstract

**Introduction:**

Remote home monitoring models were implemented during the COVID‐19 pandemic to shorten hospital length of stay, reduce unnecessary hospital admission, readmission and infection and appropriately escalate care. Within these models, patients are asked to take and record readings and escalate care if advised. There is limited evidence on how patients and carers experience these services. This study aimed to evaluate patient experiences of, and engagement with, remote home monitoring models for COVID‐19.

**Methods:**

A rapid mixed‐methods study was carried out in England (conducted from March to June 2021). We remotely conducted a cross‐sectional survey and semi‐structured interviews with patients and carers. Interview findings were summarized using rapid assessment procedures sheets and data were grouped into themes (using thematic analysis). Survey data were analysed using descriptive statistics.

**Results:**

We received 1069 surveys (18% response rate) and conducted interviews with patients (*n* = 59) or their carers (*n* = 3). ‘Care’ relied on support from staff members and family/friends. Patients and carers reported positive experiences and felt that the service and human contact reassured them and was easy to engage with. Yet, some patients and carers identified problems with engagement (e.g., hesitancy to self‐escalate care). Engagement was influenced by patient factors such as health and knowledge, support from family/friends and staff, availability and ease of use of informational and material resources (e.g., equipment) and service factors.

**Conclusion:**

Remote home monitoring models place responsibility on patients to self‐manage symptoms in partnership with staff; yet, many patients required support and preferred human contact (especially for identifying problems). Caring burden and experiences of those living alone and barriers to engagement should be considered when designing and implementing remote home monitoring services.

**Patient or Public Contribution:**

The study team met with service users and public members of the evaluation teams throughout the project in a series of workshops. Workshops informed study design, data collection tools and data interpretation and were conducted to also discuss study dissemination. Public patient involvement (PPI) members helped to pilot patient surveys and interview guides with the research team. Some members of the public also piloted the patient survey. Members of the PPI group were given the opportunity to comment on the manuscript, and the manuscript was amended accordingly.

## 1 INTRODUCTION

In recent years, there has been a shift in healthcare delivery,^1^ with services having adopted technology in different ways, including virtual consultations,^2^, ^3^, ^4^, ^5^ or remote monitoring models of healthcare.^1^, ^5^ Within remote home monitoring models, patients and carers are asked to record health readings in one place (e.g., at home), and these readings are reviewed and responded to by professionals elsewhere.^6^, ^7^ These changes in healthcare delivery potentially alter the landscape of ‘care’, as they accompany or even move away from traditional face‐to‐face care models,^8^ and instead place further emphasis on formal or informal carers providing care at a distance and reviewing readings remotely.^9^

This shift in healthcare delivery is also consistent with recent moves towards self‐management and patient activation within healthcare, whereby accountability for care has changed.^10^, ^11^, ^12^ Patients are becoming more involved in self‐management, for example, learning how to detect and manage their symptoms, and treatments, and escalation of care associated with their condition,^7^, ^13^, ^14^, ^15^, ^16^, ^17^ and healthcare tasks (e.g., managing medication, organizing care appointments, taking measurements).^18^ While some patients may welcome this,^19^ there have been concerns that self‐management places a burden on patients and families, rather than facilitating shared care.^10^, ^19^ Additionally, the effectiveness of these concepts is not fully understood yet.^12^, ^19^, ^20^, ^21^, ^22^, ^23^

The COVID‐19 pandemic further enhanced and accelerated the need for healthcare services to use technology in care delivery^5^ and escalated the need for patient self‐management. Remote home monitoring models have previously been used to provide care for chronic conditions.^24^, ^25^, ^26^ During the pandemic, remote home monitoring models were used for acute conditions such as COVID‐19, with the aim of shortening length of stay in hospital, reducing unnecessary hospital admissions or readmission and infection transmission and escalating care as needed.^27^, ^28^

Many different types of COVID‐19 remote home monitoring models were implemented throughout England. Some models referred patients from community services (e.g., GPs, hot hubs and emergency departments), known as COVID Oximetry @home.^27^ Others referred patients onto the service as early discharges from hospital, known as COVID virtual wards.^28^ See Box 1 for a brief description of services.^27^, ^28^, ^29^ According to national eligibility criteria, patients were eligible to receive these services if they had a confirmed or suspected diagnosis of COVID‐19 and were either symptomatic with COVID‐19 and aged 65 years or older, or younger than 65 years of age if clinically extremely vulnerable.

BOX 1 **Description of COVID‐19 remote home monitoring services^27^, ^28^, ^29^**

1. Patients are given a pulse oximeter, together with information and resources outlining how to use the equipment, escalation warning signs and what to do if these warning signs appear.
2. Patients measure their oxygen saturation levels using the oximeter and other readings (pulse/heart rate/temperature) regularly and record and submit these readings. Readings are shared by telephone or using a tech‐enabled method (e.g., an app on the patient's phone or computer).
3. Patients are then escalated for further care if necessary.
4. Discharge from the service is typically around 14 days.

While remote home monitoring models may reduce the need for staff to assess patients in person, they place more responsibility, commitment and workload onto patients and carers.^10^ For example, in COVID‐19 remote home monitoring services, patients and carers are expected to measure and record oxygen saturations and escalate care if readings drop below certain thresholds.^29^, ^30^ This increased responsibility may be appropriate and beneficial for some patients, but may not be suitable for everyone.^31^ Some people may be unable to meet expectations placed on them by healthcare services and experience negative impacts from treatment burden.^10^ Negative impacts may include health consequences faced by patients due to not adhering to treatment and patients' professional, social, emotional and financial situation.^18^ Different individuals may tolerate different levels of treatment burden, and it has been suggested that this needs to be assessed regularly as tolerance changes over time.^10^, ^32^ Many factors worsen treatment burden, including situational factors (e.g., travel), personal factors (e.g., beliefs and relationships) and structural factors (e.g., treatment factors and access to resources).^18^ Therefore, formal and informal support networks are needed to support patients.^7^, ^33^

Treatment burden may negatively impact on patient experience and levels of engagement. This is problematic, given that patient engagement with remote home monitoring is crucial. Patient engagement has been defined as patients understanding the information that they are given (‘receipt’) and being able to perform the required activities (‘enactment’).^34^, ^35^

While previous research indicates factors that may influence patient engagement with treatment models more generally,^7^, ^10^, ^18^, ^33^ there is a lack of research on patient experience and engagement with remote home monitoring services for an acute condition such as COVID‐19.^29^, ^30^ If patients do not engage with these services, they may be at risk of negative outcomes that the service aimed to prevent, for example, silent hypoxia (very low oxygen saturations, often without breathlessness)^36^ and/or delayed admission to hospital.^37^, ^38^ Additionally, if engagement is limited, then it is not possible to evaluate whether or not the service influences key outcome measures such as any changes in mortality or hospital use. This study addresses this gap by evaluating patient experience of and engagement with COVID‐19 remote home monitoring services.

This study aimed to explore what formal and informal support patients received as part of COVID‐19 remote home monitoring services in England, UK (COVID Oximetry@home and virtual wards models), and patient experience of and engagement with these services. This manuscript addressed the following questions:

1. What types of formal and informal support did patients receive as part of COVID‐19 remote home monitoring services? What was the burden of treatment on patients and carers in informal support roles?
2. What are patients' and carers' experiences of engaging with COVID‐19 remote home monitoring services?
3. What are the factors influencing burden of treatment and ability to engage with COVID‐19 remote home monitoring services?

## 2 METHODS

### 2.1 Design

This study used a mixed‐methods design, and included cross‐sectional survey data from patients and carers and qualitative data from semi‐structured interviews with patients and carers. A mixed‐methods study design was chosen as we sought to perform a comprehensive assessment of patients' views and experiences of these services, from a wide range of sites, and also to gain an in‐depth understanding of the factors influencing engagement with these services. The surveys helped to capture an overview of patient engagement and experience, and the interviews enabled an in‐depth understanding of experience and engagement.

This was a rapid study (data collection period: March–June 2021). Detailed methods are reported in Table 1.

**Table 1:** Detailed methods for the survey and interviews

	Survey	Interviews
Setting	This study took place in England, within NHS trusts or primary care practices/Commisioning Groups (CCGs) that implemented COVID‐19 remote home monitoring services.	
Sample—site selection	Twenty‐eight services were included in our national evaluation. 25/28 sites agreed to take part in the patient survey (reported in this manuscript). Services were sampled using a range of criteria, including the setting (primary care or secondary care), type of model (prehospital, early discharge, both), mechanism for patient monitoring (paper‐based, app, both), geographic location (across different areas of the country), timing of implementation (implemented since Wave 1 of the pandemic or recently implemented) and involvement in the evaluation with the other evaluation partners (Imperial and IAU). Sites were recruited through an expression of interest process, whereby we presented our study at local and national meetings and asked sites to express interest in participating.	A smaller sample of the overall study sites were included as case studies to conduct a more in‐depth analysis of patient experiences. Seventeen of the twenty‐five sites were selected as in‐depth case study sites using a range of criteria (setting, type of model, mechanism for patient monitoring, timing of implementation, involvement in evaluation with other partners). Four of the seventeen sites were purposively selected by NHSX for a more in‐depth analysis of patient experiences of tech‐enabled models of care; sites using different tech‐enabled platform were selected.
Sample–eligibility criteria	To participate in our survey, participants needed to be: o 18 years of age or older. o Proficient in English (or one of the following languages: Polish, Bengali, Urdu, Punjabi, French and Portuguese). o Eligible to receive COVID‐19 remote home monitoring services, and must also have been offered and received COVID‐19 remote home monitoring (national guidance: symptomatic with COVID‐19 and 65 years of age or older, symptomatic with COVID‐19, younger than 65 years of age but clinically extremely vulnerable.^27^	We aimed to interview up to six participants (patients or their carer) who had received, disengaged from or declined COVID‐19 remote home monitoring from each site. To participate in our patient or carer interviews, participants needed to be: o 18 years of age or older. o Proficient in English (or one of the following languages: Polish, Bengali, Urdu, Punjabi, French and Portuguese). o Eligible to receive COVID‐19 remote home monitoring services. o Been offered and either received or refused the service.
Measures	Patient surveys were developed specifically for this study using relevant service documentation,^27^, ^28^ theoretical frameworks^7^, ^8^, ^33^, ^39^, ^40^ and previous literature on engagement.^34^, ^35^ The survey included closed questions on the service that patients received, their experience with the service and their engagement with the service. We also asked questions about patients' experience of tech versus analogue models. Questions were followed by open ended questions to give participants the opportunity to share wider thoughts (see Appendix S1). The survey also included questions about participants' sociodemographic characteristics (gender, age, ethnicity education, employment, disability, sexual orientation, first language and geographical region).^41^, ^42^, ^43^, ^44^, ^45^, ^46^ Before use, and to ensure that the questions were appropriate, the survey was reviewed by the study clinical advisory group and reviewed by members of the study's PPI group and the public before use. The survey was amended before use (e.g., amending wording, increasing font size, adding definitions for key terms).	Interview topic guides were developed specifically for this study using relevant service documentation,^27^, ^28^ theoretical frameworks^7^, ^8^, ^33^, ^39^ and previous literature on engagement.^34^, ^35^ The topic guide included questions about their journeys of remote home monitoring, their experiences of being ill and monitored at home, experiences with escalation and discharge, their engagement with the service and recommendations for improving these models (see Appendix S2). Interviews also included questions about participants' sociodemographic characteristics (gender, age, ethnicity, education, employment, disability, sexual orientation, first language, geographical region). To determine whether questions were appropriate and relevant, we discussed the interview topic guides with our PPI members and the 70@70 nurses. The topic guides were amended accordingly.
Procedure—recruitment	Both survey options (online and paper) included prefacing information with a background to the study, potential risks, indicating voluntary participation, anonymity and a description of how the data will be used. This page also included boxes that patients/carers were asked to tick to indicate their consent to take part in the study. NHS staff distributed surveys so researchers had no access to patient information. NHS staff from participating services sent the patient survey to patients (or their carers if applicable) onboarded onto the service between 1st January 2021 and 11th June 2021. Sites chose how to disseminate the survey (post or text/email). Survey sites kept a record of the number of surveys sent out to determine the response rate. If patients were not able/willing to take part in the survey, they were given the option to ask their carer or family member to complete the survey on their behalf, reflecting on the patient's experience with the service. Patients/carers returned completed surveys directly to the study team for analysis, either electronically through REDCap or via post using pre‐paid envelopes. In addition to English, we also offered participants the opportunity to receive an information sheet and survey in six other languages (Polish, Bengali, Urdu, Punjabi, French and Portuguese).	Participants were sent an information sheet before the interview and were asked to provide written consent before taking part in the interview. At the start of the interview, researchers also confirmed verbally that participants were still happy to take part in the interview. Study coordinators at each site purposively identified a sample of participants (a range of characteristics e.g., age, gender, ethnicity), and contacted them and asked if they were happy to be approached by a researcher. The researcher then contacted them via telephone or email to discuss the study. Participants were sent information sheets and consent forms and asked to complete these before the interview (either digitally or via post). If patients were not able/willing to take part in the interview, they were asked by site coordinators if their carer (if they have one) could be approached to capture their perceptions of the patient's journey and overall experience with the service.
Procedure—data collection	Participants were approached by NHS staff at the place where they received their care (called ‘study coordinators in this manuscript), to take part in one of two ways: an online survey or a paper survey sent through the post with a free‐post envelope. Surveys were mostly distributed at discharge from the service, but some sites distributed surveys at onboarding to the service. Data collection took place between March and June 2021 (with surveys being sent retrospectively to patients who had received care from January 2021 onwards). Surveys were returned to the research team either electronically via REDCap or by posting surveys in pre‐paid envelopes to the team. Data from patient surveys sent via post were inputted into REDCap by members of the study team. All data were securely stored in the university Data Safe Haven via REDCap.	A researcher arranged a time to carry out the interview. Each site had a different lead researcher, who conducted the interviews and liaised with sites on an on‐going basis. Interviews were conducted by six researchers. Interviews were carried out via telephone or an online platform (e.g., Zoom or MS Teams) as preferred by the participant. Interviews were designed to last between 45 and 60 min. The length of interviews ranged from 05:51 to 67:38 min. Data collection for interviews was conducted between February and June 2021. All interviews were semi‐structured, audio‐recorded (subject to consent being given), transcribed verbatim by a professional transcription service (TP Transcription limited) and kept in compliance with the General Data Protection Regulation (GDPR) 2018 and Data Protection Act 2018. Interview data and transcripts were securely stored on the university Data Safe Haven. Quotes were fully anonymized before use in dissemination. Although we offered translation services for interviews, all interviews took place in English.
Analysis	The quantitative survey data were analysed using SPSS statistical software (version 25). Descriptive statistics were calculated to compare patient experiences of the service across patient groups and service models (as reported by patients and carers). In addition to the descriptive analysis presented in this manuscript, we also conducted further multivariate and univariate analyses on disparities and differences between different participant groups in relation to engagement and tech‐vs analogue modes, but these findings are presented elsewhere.^47^, ^48^ For data relating to patient experience and engagement, all cases were analysed (whether carer, patient or unknown). ‘Unknown’ cases refer to cases in which it was not clear whether the patient or carer had completed the survey. Therefore, to avoid making assumptions, we have marked these cases as ‘unknown’ but included the data relating to engagement with the service as it was still correctly completed and included reflections on their experiences. Where data were missing for specific questions, cases were excluded from the analysis and the denominator was reported. The denominators differ across questions as all questions were optional; therefore, if people decided not to complete them, this led to missing responses. Additionally, there was question routing included within our survey, which meant that not all questions were appropriate for each participant to complete. Open‐text survey data were extracted into an Excel spreadsheet and coded inductively. We extracted data from three questions: additional feedback about the service (*n* = 434 open‐text responses), how carers have supported their friend/family while they had COVID‐19 (*n* = 61 open‐text responses) and recommendations to improve the service (*n* = 200 open‐text responses). We did not receive any surveys in any other languages other than English; therefore, all analyses were conducted in English.	For patient interviews, data collection and analysis were carried out in parallel and facilitated through the use of RAP sheets as explained in Vindrola‐Padros et al.^49^ RAP sheets were developed per site to facilitate cross‐case comparisons and per population (to make comparisons between subgroups). The categories used in the RAP sheets were based on the questions included in the interview topic guide, maintaining flexibility to add categories as the study is ongoing. Research leads from each site added notes and summaries of findings to the RAP sheet following each interview, for each site. The data inputted into RAP sheets were inductively coded using thematic analysis by one researcher. Themes and subthemes were developed, discussed and agreed by the research team. We then developed a framework based on these themes and subthemes, and one researcher used this framework to extract quotes from all original transcripts.^50^ The coding framework included participants' views of the service, experiences of being referred, information received about the service and experiences performing remote home monitoring behaviours and barriers and facilitators to performing remote home monitoring behaviours. Analysis was conducted in English (as no interviews were conducted in other languages). Interview and survey data were triangulated. Interview and survey data were analysed separately initially, before being brought together to compare and contrast findings during analysis and interpretation.^51^, ^52^

Abbreviations: CCGs, clinical commisioning groups; NHS, National Health Service; PPI, public patient involvement; RAP, rapid assessment procedure.

This study was part of a larger rapid mixed‐methods evaluation of remote home monitoring for COVID‐19 patients.^53^

### 2.2 Sample

We recruited patients and carers from 25 sites (COVID‐19 remote home monitoring services delivered in National Health Service (NHS) trusts or primary care providers). We recruited sites from across six English regions, and these covered populations of <250,000 to over 1 million (see Table 2 for details). Seventeen of the twenty‐five sites participated in both surveys and in‐depth interviews; the remaining were survey‐only sites.

**Table 2:** Summary of the characteristics of included sites for the patient experience study

Characteristic^a^		Number of sites (*n* = 25)
Region		
London		5
South West		6
South East		5
North West		5
North East		2
East Midlands		2
East of England		0
Yorkshire and Humber		0
Size of the population		
<250,000		4
250,000–500,000		8
500,000–1 million		8
>1 million		5
% Urban (% rural)		
65–80 (20–35)		8
80–95 (5–20)		7
95–100 (0–5)		10
Deprivation		
% Of population in the most deprived quintile		
0–15		11
15–25		7
25–50		6
50+		1
% Of population in the least deprived quintile		
0–15		12
15–25		7
25–50		6
50+		0
Ethnicity (% of population non‐White)		
0–5		7
5–15		10
15–30		3
30–50		3
50–65		2

^a^ Sites were characterized with respect to their population size,^54^ the proportion in urban versus rural areas^55^ and the proportion in the most and least deprived areas (with respect to national quintiles).^56^ For sites based on CCG areas, we calculated these characteristics using publicly available data at the lower super output area level mapped to CCGs,^57^ while for trust‐based sites, we used data derived from inpatient Hospital episode statistics admissions during the financial year 2019/20 (Nuffield trust analysis of Hospital episode statistics admitted patient care data set, 2019/20), in addition to web searches for the trust catchment populations. Ethnicity was also calculated using publicly available data.^58^

Patients who had received COVID‐19 remote home monitoring services were recruited into the survey (aimed to recruit all onboarded patients between January 2021 and June 2021) and for the interviews (4–6 patients/carers from each of the 17 case study sites). If patients were unable to take part but wanted to participate, we invited their carer to complete the survey/interview on their behalf.

### 2.3 Measures

We developed the survey and semi‐structured topic guides specifically for this study. Questions (see Table 1) were informed by the relevant literature^7^, ^8^, ^27^, ^28^, ^33^, ^34^, ^35^, ^39^, ^40^, ^41^, ^42^, ^43^, ^44^, ^45^, ^46^ (see Appendices S1 and S2). Information sheets and the survey were available in six other languages (Polish, Bengali, Urdu, Punjabi, French and Portuguese).

The survey and interview guide were piloted with the members of the study public patient involvement (PPI) group and the general public, through the following activities: (a) workshop with the PPI group, (b) pilot interview with one PPI member and (c) survey reviewed by the PPI member and members of the public. Suggested amendments relating to accessibility and wording of questions were incorporated before use.

### 2.4 Data collection

Study coordinators working within each service distributed electronic or paper surveys to patients and carers.

Potential interview participants were approached by study coordinators from each site. If they were interested in taking part, they were contacted by a researcher, who sent them an information sheet and consent form. Participants were asked to return the consent form before the interview. Interviews were conducted by six researchers. Interviews were conducted over Microsoft Teams, Zoom or telephone.

### 2.5 Analysis

Survey data were analysed using SPSS statistical software (version 25). Descriptive statistics were used to explore patient experience and engagement (see Table 1). Open‐text survey data were extracted into an Excel spreadsheet and coded inductively.

Interview data were analysed using rapid assessment procedure (RAP) sheets (see Table 1). RAP sheets are tools that can be used to rapidly capture key findings from different data sources.^49^ The data inputted into RAP sheets were inductively coded using thematic analysis by one researcher. We developed a framework based on the themes and subthemes that we developed, and one researcher used this framework to extract quotes from all original transcripts.

Survey and interview findings were analysed separately and then triangulated to compare the consistency of the findings.^51^, ^52^

## 3 RESULTS

### 3.1 Participant characteristics

We received 1069 surveys (18% response rate) from patients (*n* = 936, 87.6%) and carers (*n* = 48, 4.5%) across 25 sites (see Table 3). In some surveys, it was unclear whether it was completed by the patient or the carer (*n* = 85, 8%).

**Table 3:** Demographic characteristics of patient and carer survey respondents

		*n* (%)
Survey respondent		
Patient		936 (87.6)
Carer		48 (4.5)
Unknown		85 (8)
Total		1069 (100)

Demographic characteristic		Patient, *n* (%)	Carer, *n* (%)
Gender (patient *n* = 920; carer *n* =45)			
Female		531 (58)	27 (60)
Male		385 (42)	18 (40)
Other/prefer not to say		4 (0.4)	0
Age (patient *n* = 923; carer *n* = 46)			
Younger than 50 years of age		195 (21.1)	13 (28.3)
50–64 years		428 (46.4)	24 (52.2)
65–79 years		256 (27.8)	4 (8.7)
≥80 years		43 (4.7)	5 (10.9)
Prefer not to say		1 (0.1)	0
Living circumstances (patient *n* = 863)			
Living alone		132 (15.3)	
Household of 2		339 (39.3)	
Household of 3		152 (17.6)	
Household of 4/5/		201 (23.3)	
Household of 6+		36 (4.2)	
Prefer not to say		3 (0.3)	
Ethnicity (patient *n* = 918; carer *n* = 47)			
White British/English/Welsh/Scottish/Irish or any other White background		836 (91.1)	38 (80.9)
Black/African/Caribbean/Black British or any other Black background		16 (1.7)	0
Asian/Asian British or any other Asian background		48 (5.2)	9 (19.1)
Mixed or multiple ethnic background		12 (1.3)	0
Any other ethnic group		2 (0.2)	0
Prefer not to say		4 (0.4)	0
Highest educational qualification (patient *n* = 914; carer *n* = 46)			
No formal qualification		146 (16)	10 (21.7)
GCSE/CSE/O level or equivalent		273 (29.9)	16 (34.8)
A level/AS level or equivalent		106 (11.6)	8 (17.4)
Degree level or higher		212 (23.2)	7 (15.2)
Other		80 (8.8)	1 (2.2)
Prefer not to say/not sure		97 (10.6)	4 (8.7)
Age completed full‐time education (patient *n* = 791; carer *n* = 28)			
15 years of age or younger		146 (18.5)	4 (14.3)
16 years		267 (33.8)	7 (25.1)
17–18 years		163 (20.6)	12 (42.9)
19–21 years		104 (13.1)	2 (7.1)
>21 years		99 (12.5)	3 (10.7)
Prefer not to say		12 (1.5)	0 (0.0)
Work situation (patient *n* = 969; carer *n* = 45)^a^			
Working full time/self‐employed		396 (41.9)	17 (35.4)
Working part time		128 (13.5)	6 (12.5)
Student in higher education		2 (0.2)	0
Unemployed		18 (1.9)	3 (6.3)
Homemaker/full‐time carer		40 (4.2)	4 (8.4)
Retired		274 (29)	9 (18.8)
Furloughed		15 (1.6)	0
Not in work due to poor health or disability		65 (6.9)	5 (10.4)
Other/prefer not to say		31 (3.3)	1 (2.1)
Sexual orientation (patient *n* = 919; carer *n* = 44)			
Straight/heterosexual		858 (93.4)	41 (93.2)
Gay or lesbian		13 (1.4)	0
Bisexual		5 (0.5)	0
Other/prefer not to say		43 (4.7)	3 (6.8)
English as first language (patient *n* = 925; carer *n* = 43)			
Yes		852 (92.1)	35 (81.4)
No		66 (7.1)	8 (18.6)
Prefer not to say		7 (0.8)	0
Day‐to‐day activities limited by a health problem or disability (patient *n*= 920; carer *n* = 46)			
Limited a lot or a little		351 (38.1)	20 (43.4)
Not limited at all		482 (52.4)	17 (37)
Prefer not to say/not sure/not applicable		87 (9.4)	9 (19.6)
Deprivation score^b^ (patient *n* = 767; carer *n* = 37)			
D1 or D2 (most deprived)		182 (23.7)	13 (35.1)
D3 or D4		137 (17.9)	5 (13.5)
D5 or D6		149 (19.4)	7 (18.9)
D7 or D8		161 (21)	9 (24.3)
D9 or D10 (least deprived)		138 (18)	3 (8.1)
Relationship with patient (carer *n* = 42)			
Spouse or partner			24 (57.1)
Son or daughter			11 (26.2)
Other			7 (16.7)

^a^ Respondents able to select more than one option.

^b^ Deprivation scores are based on postcode, and are reported using deciles instead of quintiles (as with the site characteristics) to check that participant characteristics were representative across these 10 deciles.

We conducted 62 interviews with patients (*n* = 59) and carers (*n* = 3) across 17 sites (see Table 4 for demographics). However, we were unable to recruit any participants who declined the service or disengaged from the service.

**Table 4:** Demographic characteristics of patients and carer interview respondents

Demographic characteristic		Patient, *n* (%)^a^	Carer, *n* (%)
Patient or carer who took part in the interview		59 (95%)	3 (5%)
Gender			
Female		31 (50%)	3 (100%)
Male		31 (50%)	0
Age			
Younger than 50 years of age		8 (13%)	2 (67%)
50–64 years		31 (50%)	
65–79 years		21 (34%)	1 (33%)
≥80 years		2 (3%)	
Living circumstances			
Live alone		5 (8%)	
Household of 2		36 (58%)	2 (67%)
Household of 3		11 (18%)	
Household of 4–5		9 (15%)	
Household of 6+		1 (2%)	1 (33%)
Home ownership/renting			
Own home outright		26 (42%)	1 (33%)
Own home with mortgage		16 (26%)	1 (33%)
Own home (not specified)		3 (5%)	
Rent from local authority/house association		9 (15%)	
Rents privately		6 (10%)	
Other		2 (3%)	1 (33%)
Ethnicity			
White British/English/Welsh/Scottish		50 (81%)	
White Irish			
Any other white background			
Black/African/Caribbean/Black British		3 (5%)	
Asian/Asian British		7 (11%)	2 (67%)
Missing		1 (2%)	1 (33%)
Not enough information		1 (2%)	
Age completed full‐time education			
15 years of age or younger		13 (21%)	
16 years		21 (34%)	
17–18 years		16 (26%)	
19–21 years		5 (8%)	
>21 years		5 (8%)	1 (33%)
Not known		2 (3%)	2 (67%)
Highest educational qualification			
No formal qualification		14 (23%)	
GCSE/CSE/O level or equivalent		21 (34%)	
A level/AS level or equivalent		5 (8%)	
Degree level or higher		14 (23%)	2 (67%)
Other		7 (11%)	
Not sure		1 (2%)	1 (33%)
Work situation			
Working full time		25 (40%)	2 (67%)
Working part time		1 (2%)	
Self‐employed		2 (3%)	
Not working		1 (2%)	
Homemaker		2 (3%)	
Retired		23 (37%)	
Furloughed		1 (2%)	
Not in work due to poor health or disability		7 (11%)	
Not sure		0 (0%)	1 (33%)
Sexual orientation			
Straight/heterosexual		62 (100%)	3 (100%)
English as first language			
Yes		54 (87%)	3 (100%)
No		7 (11%)	
Not specified		1 (2%)	
Day‐to‐day activities limited by a health problem or disability			
Yes limited a lot		6 (10%)	
Yes limited a little		6 (10%)	
Limited (but not specified how much)		4 (6%)	
No, not limited at all		46 (74%)	2 (67%)
Not known			1 (33%)
Relationship with patient			
Spouse or partner			1 (33%)
Son or daughter			2 (67%)

^a^ 59 Patients took part in the interviews, but we have demographic characteristics for 62 patients as carers reported patient demographics too.

Most patients (70% *n* = 749/1069 survey and 71% *n* = 44/62 interview participants) were referred to the service via community methods (see Table 5). Patients and carers reported using a range of methods to record and report their readings to the service, including analogue (paper and phone) (49%, *n* = 522/1069 survey and 31% *n* = 19/62 interview participants) and tech‐enabled methods (51% *n* = 547/1069 survey and 44% *n* = 27/62 interview participants) (see Table 5).

### 3.2 What types of formal and informal support did patients receive as part of COVID‐19 remote home monitoring services?

Below, we describe a summary of survey findings (see Table 6) and interview findings relating to formal and informal support.

**Table 5:** Summary of patients' remote home monitoring pathway and method of recording and reporting

		Survey participants (*n* = 1069), *n* (%)	Interview participants (*n* = 62), *n* (%)
Pathway			
COVID Oximetry @home (referred prehospital via community methods)		749 (70%)	44 (71%)
Virtual ward (referred via early discharge from hospital)		168 (16%)	13 (21%)
Both		N/A	2 (3%)
Unknown		152 (14%)	2 (3%)
Not applicable		N/A	1 (1%)
Method used to record and report readings			
Analogue (paper and phone)		522 (49%)	19 (31%)
Tech‐enabled (such as text, app, weblink or automated phone)		547 (51%)	27 (44%)
Combination of tech‐enabled and analogue		N/A	14 (23%)
Not known		N/A	1 (2%)
Not applicable		N/A	1 (2%)

**Table 6:** Summary of survey findings relating to participants' experience of and engagement with the remote home monitoring services

Survey question and response		Percentage of survey participants, *n* (%)	Percentage range across sites
Frequency of contact with staff member (*n* = 1060)			
Several times a day		169 (16%)	0%–45%
Once a day		276 (26%)	7%–91%
Several times a week		270 (25%)	0%–62%
Once a week		139 (13%)	0%–31%
Less than once a week		142 (13%)	0%–27%
Not at all		64 (6%)	0%–27%
Remote home monitoring activities that patients reported doing (*n* = 1069)^a^			
Using the oximeter		1014 (95%)	81%–100%
Completing a diary		555 (52%)	11%–89%
Providing readings over the phone		498 (47%)	19%–93%
Providing readings via text		309 (29%)	0%–74%
Recording readings in a digital app		264 (25%)	0%–89%
Providing readings via email		15 (1%)	0%–5%
Seeking further help due to readings being lower than the recommended threshold		344 (32%)	18%–71%
Checking over readings for issues		215 (20%)	7%–41%
Support for remote home monitoring activities			
Having someone to help use the oximeter when needed (*n* = 1058)			
Yes		782 (74%)	50%–100%
No		169 (16%)	0%–44%
Not applicable		107 (10%)	0%–22%
Support taking and recording readings if needed (*n* = 1057)			
Yes		666 (63%)	44%–90%
No		190 (18%)	7%–33%
Not applicable		201 (19%)	0%–38%
Experience engaging with service activities			
Understanding the information they were given (*n* = 1040)			
Easy/very easy		970 (93%)	71%–100%
Neutral		57 (5%)	0%–29%
Difficult/very difficult		13 (1%)	0%–9%
Monitoring using the oximeter (*n* = 1049)			
Easy/very easy		1022 (97%)	83%–100%
Neutral		18 (2%)	0%–9%
Difficult/very difficult		9 (1%)	0%–8%
Recording readings (*n* = 949)			
Easy/very easy		913 (96%)	75%–100%
Neutral		21 (2%)	0%–25%
Difficult/very difficult		15 (2%)	0%–4%
Providing readings to the remote home monitoring team (*n* = 1010)			
Easy/very easy		979 (97%)	88%–100%
Neutral		21 (2%)	0%–13%
Difficult/very difficult		10 (1%)	0%–4%
Seeking further help (if applicable) (*n* = 857)			
Easy/very easy		738 (86%)	60%–97%
Neutral		75 (9%)	0%–22%
Difficult/very difficult		44 (5%)	0%–23%
Challenges experienced with service activities (*n* = 1069)^a^			
Using the oximeter		34 (3%)	0%–9%
Recording readings in an app or diary		27 (3%)	0%–9%
Providing readings to the service		32 (3%)	0%–13%
Contacting healthcare professionals when needed		56 (5%)	0%–36%
Seeking further help		57 (5%)	0%–36%
Returning the oximeter		136 (13%)	0%–36%
Other		42 (4%)	0%–14%
Discussion and resolution of problems			
Discussed problems with remote home monitoring team (*n* = 249)		87 (35%)	0%–100%
Had problems resolved (*n* = 232)		76 (33%)	0%–100%
Did not have problems resolved (*n* = 232)		126 (54%)	0%–100%

^a^ Respondents were able to select more than one response option.

#### 3.2.1 Formal support from staff

The ‘care’ on offer differed across sites and patients, with variation in the type and frequency of monitoring offered by services.

Responses from the patient survey indicated that the frequency with which patients had contact with a member of staff varied, but that most patients and carers had contact either once a day (26%, *n* = 276/1060) or several times a week (25%, *n* = 270/1060). A few patients and carers reported not speaking to staff at all (see Table 6).

This was supported by interview findings, which indicated that the frequency of taking and communicating readings to the service ranged from once a day to more than three times a day. Findings indicate that patients are supported by staff throughout different stages of the service, including providing information, monitoring (e.g., phone calls if patients and carers forget to submit readings and in some cases face‐to‐face visits to take readings), escalating care (e.g., providing advice on whether to seek help, calling ambulances for patients), signposting and comfort and reassurance.

#### 3.2.2 Burden of treatment on patients and carers in informal support roles

Survey findings indicated that almost all patients used an oximeter to record readings when receiving the service (95%, *n* = 1014/1069). Many patients reported completing a diary (52%, *n* = 555/1069) and providing readings over the phone (47%, *n* = 498/1069) or using technology‐enabled methods (e.g., text 29%, *n* = 309/1069). Escalation‐related behaviours were reported less frequently by patients, with only a third of patients reporting seeking further help due to readings being lower than recommended thresholds (32%, *n* = 344/1069), and only a fifth of patients reporting checking their readings for issues (20%, *n* = 215/1069) (see Table 6).

Many patients were supported by family and friends to engage with the service. A quarter of survey respondents needed help to use equipment (25%, range 11%–50% across sites), and more than half of the interview participants were supported by family members.

Most patients and carers reported having informal support to help them use the oximeter and support with taking and recording readings. Only a small proportion of participants reported that they did not need support using the oximeter (10%, *n* = 107/1058) or taking and recording readings (19%, *n* = 201/1057) (see Table 6).

Qualitative findings highlighted that family and friends provided support with the following activities: support submitting readings or communicating readings over the phone, support with monitoring, support collecting oximeters, support writing down readings and contacting and taking calls from the service, translation support or using the app. Additionally, other patients reported that their family members and friends provided comfort and reassurance, support as and when needed, support and advice at a distance and domestic care. Open‐text survey responses indicated that many carers provided full‐time care for their family member/friend while on the service. Some carers were family members or friends who moved in to provide support. Others made regular telephone calls to check in on their family member.

However, not all patients had support with using the oximeter (16%, *n* = 169/1058) or taking/recording readings (18%, *n* = 190/1058). Some patients did not have support due to their family members having COVID‐19.

### 3.3 What are patients' and carers' experiences of engaging with COVID‐19 remote home monitoring services?

Patients mostly had positive views of the service. 93% (*n* = 970/1045) of survey respondents rated the service as excellent or good, 90% (*n* = 923/1028) of respondents found the service helpful and 91% (*n* = 944/1037) would recommend the service to their family and friends.

Findings indicated that most patients and carers found the service reassuring and supportive (91% [*n* = 946/1040] of survey respondents). Qualitative findings indicated that patients and carers valued the human contact with staff and found it reassuring due to having someone watching over them, particularly for those who were living alone, had no support nearby or had existing conditions.

*Because it's obviously keeping an eye on you, isn't it really? And I was getting the phone calls every day. How are you feeling?, […]. But someone who was on their own, who had no‐ who was living on their own, you know, it's a bit of a lifesaver isn't it?*. (Site A, interviewee 4)

A minority of patients and carers felt that there were gaps in the service, and it was not holistic. Some felt that the service was narrowly focused on managing known symptoms of COVID‐19, which did not always suit those with other symptoms, health conditions or who required wider support. A few patients reported feeling that the service was isolating and unsupportive (e.g., they only received a call about the oximeter dropoff/return, but not for monitoring).

Most patients and carers felt that the care provided was appropriate and preferred to be at home instead of being in hospital (given the pandemic context). Reasons for preferring home over hospital included freeing up space for others in need, being familiar with your environment, fears of going to hospital during a pandemic, communication barriers in hospital, being able to work and perceptions that home monitoring was a suitable care package for those with more minor symptoms of COVID‐19. However, some patients and carers spoke about preferring to be in the hospital rather than at home, to feel more secure, feeling scared and wanting to be seen face to face.

*And I didn't feel too embarrassed that I was using up valuable resources because I thought, Well I'm sitting here at home, there's no reason for me to go in Hospital, bother anyone and waste people's time*. (Site N, interviewee 3)

A minority of patients and carers spoke about how the service was the only available care and that they would have liked to have received care from other healthcare professionals such as their GP in parallel. Many patients and carers were not aware of the service before referral.

Some patients and carers also spoke about how the service helped them to monitor their own improvement and that it potentially improved their outcomes.

Patients and carers reported very positive views of the workforce and that they were helpful and put patients at ease, and were professional and potentially even lifesaving. Continuity of staff was thought to be important.

A few patients had negative experiences with individual staff members, for example, that they were dismissive, did not recognize that they needed help, were not interested or lacked clinical expertize to support patients or answer their queries.

### 3.4 What are the factors influencing burden of treatment and ability to engage with COVID‐19 remote home monitoring services?

Findings indicated that patients and carers generally found it easy or very easy to engage with the service and the resulting activities, including understanding information, monitoring using the oximeter, recording readings and providing readings and escalating care (see Table 6). Most survey respondents indicated that they did not experience problems with the service (72%; *n* = 771/1069) and did not report barriers to engagement with the service (80%, *n* = 858/1069).

Engagement with service activities was not without challenges, with some patients and carers reporting issues with the information provided or needed further information. Some patients found monitoring difficult due to other health conditions, or that monitoring made them feel worried. Some patients and carers wanted more support or found recording burdensome. Finally, some issues related to escalating care were identified in the interviews. The uncertainty of COVID‐19, perceptions of hospital as a frightening place and uncertainty around interpretation of readings and thresholds meant that some patients and carers were hesitant to self‐escalate their care, waited for a member of staff to advise them to escalate their care or reported not wanting to go to hospital or seek further support even when advised to by staff members.

*Really I should have probably rung when the readings were that bad but I didn't. […] And when I did send them through they said, ‘No, get to the doctors now'*. (Site C interviewee 6)

The most frequent challenges reported within the survey were returning the oximeter, contacting healthcare professionals when needed and seeking further help (see Table 6). While many survey respondents discussed problems with the team (35%, *n* = 87/249) or had their problems resolved (33%, *n* = 76/232), over half of these participants said that problems had not been resolved (54%, *n* = 126/232).

Findings from the surveys and interviews indicated three overarching themes that influenced burden of treatment and patients' ability to engage with COVID‐19 remote home monitoring services: (i) patient factors, (ii) wider support and resources and (iii) factors relating to the service (see Table 7 for details of example findings for each theme and subtheme and example quotes).

**Table 7:** Summary of survey and interview findings for factors influencing engagement

Theme	Summary of survey and interview findings for subthemes	Example quotes
Patient factors	*Knowledge*: A majority of survey participants felt that knowing what to do helped them to engage with the service (55%, *n* = 583/1069). This was supported by interview findings that indicated that participants having the appropriate knowledge helped them to use the oximeter, monitor/record/communicate readings and escalate care. However, interview findings indicated that knowledge was a barrier for some participants (e.g., relating to understanding/interpreting information and equipment, language barriers, not knowing how to fill out diary/complete readings/escalate care or when to call for help). *Memory*: Forgetting to do the readings was a barrier to engaging with the service for some survey (4%, *n* = 37/1069) and interview participants. However, phone calls helped participants to do readings, and some participants wrote readings on post‐it notes to facilitate memory. *Physical health*: A majority of survey respondents felt that their own health helped them to engage (54%, *n* = 581/1069). However, within the interviews, many barriers relating to physical health were identified, including feeling too poorly/not in the right frame of mind, sleeping a lot, having health conditions that made it difficult to monitor, difficulties hearing, difficulties getting to the telephone and difficulties with eyesight. These barriers were also reported within the survey (4%, *n* = 39/1069 reported own health as a barrier, and some participants wrote in open‐text findings that they felt too poorly to engage). *Attitudes towards the service and behaviours*: Survey and interview participants reported knowing why the service was important (e.g., 60%, *n* = 637/1069 survey respondents) and wanting to engage with the service (46%, *n* = 491/1069), and these positive views helped them to engage with the service. However, some participants reported a lack of interest in monitoring/recording, or views that monitoring did not help or made them anxious if the reading was low. Additionally, barriers to seeking further support/escalating care were identified, including worries about going to hospital (due to COVID‐19, lack of support, difficulties communicating). *Time*: A fifth of survey participants felt that having time helped them to engage with the service (20%, *n* = 217/1069). Interview findings supported this by highlighting the importance of developing a routine. However, a few participants did not have enough time (e.g., those working from home).	‘Well I mean. It's quite straightforward isn't it. You just put it on your finger and let it settle down and read the figures off. Pulse‐pulse and oxygen levels. So no, I didn't find it complex at all’. (Site C, interviewee 2) ‘Really they just, in the main I was quite poorly, in fact I would say I was really poorly, it's the only time I've thought I was going to die in my life […] so really in the main my husband dealt with them, I couldn't really be remotely bothered with them if I'm honest and I can't remember what they told me, I don't think they told me a lot’. (Site I, interviewee 3) ‘But there are sometimes, I must admit, sometimes it makes you feel a little bit anxious [.] but then you can leave it – because you want to get things all right – but towards the end of the day it is so worth it – it is 100% worth it to have the oximeters reading every day to know; to understand where you are […] but I still guarantee that 100% it is a very good idea’. (Site A, interviewee 1) ‘Just really, they encouraged me to ring an Ambulance if I needed it. And I wasn't ringing them, because I felt like I was wasting their time, or whatever. I didn't want to, because I was worried they might want to take me in’. (Site B, interviewee 4)
Wider support and resources	*Support from staff/service*: Survey and interview participants spoke about how support from healthcare professionals helped them to engage with the service (46%, *n* = 488/1069). Interview findings highlighted that support helped to understand information, helped with monitoring, obtaining equipment, recording, communication and escalating care. Support was reassuring. However, a small number did highlight that they did not have enough support from healthcare professionals or that they could not get through to a member of the team. *Support from family/friends*: A quarter of survey respondents (25%, *n* = 266/1069) felt that support from family and friends helped them to engage. Interview findings echoed this and highlighted that support from family and friends helped with understanding information, collecting the oximeter, doing the monitoring, recording and communicating readings and escalating care. However, a small number of participants reported that lack of support from family/friends was a barrier to engagement. *Accessibility and availability of materials*: The amount of information received was sometimes reported as a barrier in the interviews (e.g., too much, too little or contradictory and confusing information). *Equipment*: Some participants already had their own equipment, which facilitated engagement, and a few participants reported not having the right equipment (e.g., faulty oximeters/not having thermometers) *Technology*: Some participants felt that reminder texts or alerts from an app helped them to engage, and that the technology was easy to use. However, other participants experienced difficulties with the app, oximeter and technology systems.	‘The nurse was very good, can't praise her really high enough. She was a friendly voice to speak to. Fair enough, you know, I've got a bit of a support system, but for somebody who hasn't got that much of a support system around them, I think that friendly voice would go a long way just to, you know, easing their minds’. Site D, interviewee 5 ‘And then near the end when I was getting a bit complacent, I was sort of almost well, a couple of times I didn't put them in and they would phone and say, “Are you okay? You've not submitted you reading.” So that was just really supportive, and I said it certainly reassured me’. (Site M, interviewee 1) ‘So my dad was initially involved in I think it was nine days, so the first nine days he took full care of mum to be honest clinically I was involved in a lot of the calls because I think my dad's getting quite stressed. […] so yes, he did the physical side of it. He would do the observations. And then he'd call me first thing in the morning, or he'd drop a text to say these are the observations. I'd call and have a quick chat, knowing the nurse was going to call us. So I guess it was a bit of a joint effort between us’. (Site F, interviewee 6)
Service factors	*Monitoring characteristics*: Participants identified barriers relating to inconsistency of call timing, amount of calls, not being able to see progress and frequency of monitoring and recording. *Service characteristics*: Some participants felt that there were problems relating to delays in enrolment, limited hours of service and not being able to continue monitoring following discharge. *Scope of service*: Scope of service was a barrier to escalation as some participants did not know whether to ring to ask for help. Some participants reported that the service was not holistic (did not cover all symptoms of COVID). *Availability of treatment*: Some participants mentioned problems contacting their GP, and inability to receive oxygen in their own home if needed as barriers.	‘I found, to start with I found the text messages useful but the longer they went on the more irritating. I was, I felt like I was chained to the phone and you know and to my equipment. So three times a day is, I know that's necessary to start with but I just felt that maybe twice a day after that might have been better’. (Site B, interviewee 3) ‘I think somebody should maybe discuss some of the other things. To me, I got the impression that as long as I as breathing and my oxygen levels were reasonable, that is all they were interested in. Where there were other things that I was a bit concerned about which I don't think were discussed unless I brought it up’. (Site J, interviewee 1)

#### 3.4.1 Patient factors

Knowledge, memory, physical health, attitudes towards the service and having time to complete the required tasks influenced engagement (see Table 7). Interview findings indicated that patients in poorer health (e.g., due to COVID‐19, other health conditions) found it harder to engage with the service. For example, many participants spoke about feeling too unwell due to COVID‐19 (often during the first few days of the service) and therefore they were unable to engage with monitoring behaviours such as taking and recording readings. Some patients and carers spoke about having other health conditions that made it difficult to engage with monitoring behaviours (e.g., hearing and eyesight difficulties). Patients and carers who felt that they had sufficient knowledge about what they needed to do found it easier to engage (e.g., 55% (*n* = 583/1069) of survey respondents felt that knowing what to do helped them to engage with the service). On the other hand, a lack of knowledge of how to complete the activities (e.g., a lack of knowledge of how to escalate care or what the thresholds for escalating care are) limited engagement.

#### 3.4.2 Wider support and resources

Support from staff/service, support from family members/friends, accessibility and availability of materials, equipment and technology influenced engagement (see Table 7). For example, support from staff members (e.g., 46% [*n* = 488/1069] of survey respondents) and family/friends (e.g., 25% [*n* = 266/1069] of survey respondents) was crucial in helping many patients to use the service.

#### 3.4.3 Service factors

Monitoring characteristics, service characteristics, scope of service and availability of treatment influenced engagement (see Table 7). For example, some participants felt that the inconsistent timing of calls was a barrier and some felt that calls were too frequent, whereas others felt that they were not frequent enough. Additionally, some patients and carers felt that the scope of the service was a barrier to engagement, in that it did not cover wide symptoms of COVID‐19 and was not holistic.

## 4 DISCUSSION

### 4.1 How findings relate to previous research

Findings indicated that patients can engage with remote home monitoring services, even when experiencing acute illnesses (e.g., COVID‐19). However, many patients required formal input from staff and informal support from family and friends to complete the necessary tasks (e.g., monitoring oxygen saturations). Patients and carers had positive experiences receiving remote home monitoring. The human contact from staff provided patients and carers with reassurance and patients reported that the service was mostly easy to engage with. We identified some challenges to engagement (e.g., hesitancy to seek further support or self‐escalate care when readings dropped below a certain threshold). Patients' ability to engage with the service was conditional on a range of factors, including having support from family/friends and staff, being in good health and receiving clear instructions on what they needed to do and how to do it, and the level of commitment from patients while on the service.

Earlier studies have explored types of remote home monitoring, and implementation of and cost of remote home monitoring models for COVID‐19.^29^, ^30^ Yet, there was little research on patient experiences of remote home monitoring services when delivered during a pandemic in the context of pressured health services and concerned patients. These findings extend earlier findings by highlighting patients' and carers' positive views of the service and challenges and concerns relating to engagement with remote home monitoring for acute conditions.

Previous research outlines concerns relating to remote care and telemedicine and the loss of interpersonal dimensions involved in caring relationships.^9^ Findings extend the evidence base by suggesting that care does not need to take place face to face for patients to feel reassured and supported. Patients largely felt that care provided at a distance was appropriate and that they were being monitored. This is consistent with previous research indicating that technology may support closer contact with professionals.^9^ However, findings may have been affected by the pandemic context in that data were collected during the height of Wave 2 of the pandemic; therefore, patients may have been more likely to accept remotely delivered services to help minimize risk to themselves, family members and staff. Patients and carers may feel differently about remote home monitoring and care delivered at a distance in non‐pandemic contexts.

New models of healthcare such as COVID‐19 remote home monitoring services sought to change the traditional model of in‐person care. Instead, within these models, staff engage with patients to share the care burden while equipping patients and carers to self‐monitor and manage care in the absence of staff members. Our findings demonstrate that concepts of treatment burden and difficulties engaging with healthcare demands^10^, ^18^, ^32^ also apply to remote monitoring models for COVID‐19. Some patients reported problems engaging with remote home monitoring services for a range of reasons, including feeling too poorly, not having enough knowledge of what to do and lack of support from staff and/or family/friends. Others reported the necessity of support from their family/friends when engaging with the service. This extends knowledge by showing that social networks may undertake self‐monitoring tasks on behalf of (potentially very poorly) patients in addition to helping patients cope with burden of treatment^10^ and self‐management of conditions.^7^, ^59^ Those who feel more poorly (either due to COVID‐19 or existing conditions) may require more formal or informal support to manage care. Yet, this increases the caring burden for family/friends. This finding also raises concerns regarding appropriateness of care for those who do not have informal support networks in place.

This manuscript builds on earlier research by providing a nuanced interpretation of the factors that influenced engagement with remote home monitoring services for acute conditions such as COVID‐19. Our finding that many non health‐related factors influence engagement is consistent with other studies in a range of other conditions and interventions.^7^, ^18^, ^60^, ^61^, ^62^, ^63^ Our findings add to prior knowledge by demonstrating that many factors were exacerbated due to the acute nature of COVID‐19 and policy factors surrounding COVID. For example, physical health factors limiting engagement may be worsened by the acute nature of COVID‐19 and the severity of symptoms that some patients faced, thus affecting a patient's ability to engage. Furthermore, policy regulations and lockdown restrictions imposed within the UK may have meant that physical social support available to patients may have been limited by members of their support networks living elsewhere. As COVID‐19 is easily transmissible, social distancing recommendations were in place; therefore, many patients were distancing from family members living in the same space. Findings demonstrate that despite difficulties imposed by COVID‐19, social networks were crucial for many patients in facilitating engagement with the service and ensuring that care needs were met. This highlights the need for alternative support where necessary (particularly for those living alone or those who are socially isolated). The reliance on informal support networks has implications for burden of treatment and may not be appropriate for all individuals. However, it is important to note that some patients were able to engage with the service (as they had manageable symptoms and felt comfortable with the task). This indicates that different levels of remote monitoring support are needed for different individuals. This supports previous research indicating that the success of telehealth services including remote home monitoring may rely on the fit between individuals' needs and services.^9^, ^33^ These findings support previous theoretical frameworks indicating that social, political and technical contexts influence engagement.^7^, ^8^, ^33^, ^39^

Previous research has explored concepts of self‐management,^7^, ^33^, ^60^ engagement^61^ and treatment burden^18^ in chronic conditions, but little research had been conducted on remote home monitoring and self‐management in acute conditions such as COVID‐19, in which care needs to be urgently escalated in an efficient and time‐sensitive manner. Findings extend earlier work by demonstrating the challenges of remote home monitoring models for acute conditions. For example, due to the uncertainty of COVID‐19, perceptions of hospital being a frightening place and uncertainty around readings and thresholds, some patients were hesitant to self‐escalate care and in many cases, patients waited until advised by staff to escalate care. This finding indicates that in situations where there is a need for timely escalation, but concerns around infection transmission from going to hospital, it may be suitable to have formal support from staff members. Together, staff and patients can collaboratively decide when to seek further help, rather than placing responsibility onto patients. This finding contrasts with recommendations within the national standard operating procedures for COVID‐19 remote home monitoring services,^27^, ^28^ which indicate that pathways should encourage patients to self‐escalate care.

### 4.2 Strengths and limitations

Integration of mixed‐methods data helped to provide in‐depth perspectives on experiences of, and engagement with, COVID‐19 remote home monitoring services. A large team of researchers (from a range of disciplines, with extensive expertize in qualitative and quantitative methods) was involved, thus strengthening the interpretation of findings. Findings were shared with clinical and academic stakeholders. Our study sampled a large range of sites with a range of characteristics, thus enhancing the generalizability of the findings.

Compared with patient onboarding data, our patient sample was underrepresentative of some groups (e.g., older patients, Black, Asian and minority ethnic communities and most deprived) and overrepresentative of other groups.^64^ The response rate for the survey was fairly low (17.5%). Additionally, we were unable to recruit interview or survey participants who had declined the service, dropped out from the service and those who were unable or did not want to take part in surveys and interviews. Therefore, findings may not be representative of all patient groups and experiences.

While we did include carers within our sample, the focus of our research was on patient experiences of remote home monitoring services. Therefore, it is possible that we have not captured carers' experiences in detail. However, some carers shared their own experiences during the interviews and in responding to the survey.

### 4.3 Implications

Burden of treatment may not only affect those with multimorbidity or chronic conditions but can also affect those with acute conditions. Findings indicate that remote monitoring may increase treatment burden for some patients and families.

COVID‐19 remote home monitoring services aimed to target patient groups at higher risk from COVID‐19, and yet, many of these groups appear more likely to report difficulties in engagement with these services, for example, older patients and patients with health problems. Remote monitoring may not be appropriate for everyone (e.g., those without support). Services need to gauge a person's support network and any concerns surrounding remote home monitoring when assessing eligibility for these services. Services must then tailor the healthcare offer to enable patients to engage (e.g., providing further support for those from at‐risk groups or who do not have informal support, or linking patients with care networks if needed). All patients should be provided contact details to contact the service, should problems arise. Face‐to‐face support (e.g., for monitoring) from staff and families has implications for infection transmission.

Our findings may have implications for remote home monitoring services more generally. Service developers should consider the type of condition when designing pathways. For example, services for acute conditions may require support from staff to ensure that patients are escalated for further care as necessary. Services must plan logistics for delivery and collection of equipment, ensure sufficient information provision and that patients know what they need to do and that they feel able to engage with the service. Some patients felt that the service offered was too narrow and does not consider wider social, emotional or condition‐related needs. Service adaptations may be necessary for those receiving remote home monitoring for acute conditions in addition to care for other chronic conditions. Our findings provide some tangible recommendations from the patients' perspective on how to improve remote home monitoring services (see Appendix S3), many of which support wider themes reported in the patient experience literature (e.g., the importance of information provision).^65^, ^66^ Our findings, together with our wider findings on effectiveness,^67^, ^68^ cost,^64^ implementation,^64^ workforce,^64^ disparities^47^ and mode,^48^ may be helpful in the development of wider remote home monitoring services, for example, virtual ward services that are currently being rolled out across England for a range of conditions.^69^, ^70^

### 4.4 Future research

Further research is needed to explore the experiences of those who decide not to use remote home monitoring services or disengage from these services. Further research should explore the burden of treatment for chronic conditions compared with acute conditions. Additionally, it would be helpful to further explore which groups are able to tolerate burden associated with remote home monitoring pathways and the impact of treatment burden from informal caring responsibilities on families.

## 5 CONCLUSIONS

COVID‐19 remote home monitoring services place a large responsibility on patients and carers in relation to monitoring and escalating care. While patients and carers found the service reassuring and a positive experience, many factors influenced their ability to engage with the service. This indicates that the service may be conditional on a range of factors relating to the patient (e.g., knowledge and memory), their support and resources (e.g., support from family, friends and staff) and service factors (e.g., scope of the service and frequency of monitoring).

## AUTHOR CONTRIBUTIONS

All authors were responsible for the study conception, design and data collection throughout the study. Holly Walton, Cecilia Vindrola‐Padros and Nadia Crellin led the data analysis. Holly Walton and Cecilia Vindrola‐Padros drafted the manuscript with contribution from all authors. All authors commented on drafts of the manuscript and approved the final version. Naomi J. Fulop was the principal investigator for the study.

## CONFLICT OF INTEREST

The authors declare no conflict of interest.

## ETHICS STATEMENT

For this evaluation, the research was divided into two separate protocols. A protocol covering effectiveness, cost and staff elements received ethical approval from the University of Birmingham Humanities and Social Sciences ethics committee (ERN_13‐1085AP39) and was categorized as a service evaluation by the HRA decision tool and UCL/UCLH Joint Research Office. The patient experience study reported in this manuscript (survey and case study interviews) was reviewed and given favourable opinion by the London‐Bloomsbury Research ethics committee (REC reference: 21/HRA/0155).

## Supporting information

Supporting information.

Supporting information.

Supporting information.

## Data Availability

The data that support the findings of this study are available on request from the corresponding author. The data are not publicly available due to privacy or ethical restrictions.
