# Supplementary material for: Patients' experiences of, and engagement with, remote home monitoring services for COVID‐19 patients: A rapid mixed‐methods study
Source: Health Expect. 2022 Jul 7;25(5):2386–404. doi: 10.1111/hex.13548 (PMC9349790; doi:10.1111/hex.13548)
Supplement: Supplementary file 2 — Supporting information. [file HEX-25--s002.docx]

**Appendix 2. Interview topic guide**

**PATIENTS**

**INTRODUCTION**

The interview should last between 30 and 60 mins, depending on how much you would like to say. We will ask questions about how you found the experience of monitoring and recording your COVID symptoms at home, and any further advice you received – we call this “COVID care at home”. We will feedback the results of this evaluation to local and national NHS and public health services, and results will be made available to the general public too.

If you do not want to answer a question, you do not have to, and if you feel uncomfortable or tired we can stop the interview at any point. Let us know if you’d like a break or to come back later. We can also carry out the interview in two halves if that is easier for you to manage. Have you got any questions before we start?

**Questions for those who have received CO@h**

| **Main question** | **Follow up questions (prompts)** |
| --- | --- |
| 1. Please tell me a bit about yourself, | - Do you live by yourself or with others? - How long have you lived in your neighbourhood? - Do you have any family or friends living close by? - Is English your first language? If N, ask what is their first language. |
| **FINDING OUT ABOUT THE SERVICE**   1. Can you tell me the story of how you ended up being referred to COVID care at home? | - 1. How were you referred to the service?   2. Who did you speak to and when? (virtual/face to face? How were you involved in the assessment process?) *(for assessment/triage)*   3. When you were first told about the service, how was it described to you?   4. What were your first impressions of COVID care at home? *(prompt about whether they found it reassuring or not)*      1. Positive impressions?      2. Did you have any concerns/worries? |
| **DESCRIBING COVID CARE AT HOME**  3. COVID care at home is carried out slightly differently in different parts of the country. Please can you tell me about what receiving COVID care at home has involved for you? | a. What equipment were you given?  b. How/when was the pulse oximeter delivered to you? Can you tell me how it works?  c. What symptoms did you have to monitor? For each, ask how often.  d. How did you have to record your symptoms? (e.g. paper, digital app, telephone line)  e. Were you offered a choice in how you recorded your symptoms?  f. Who did you speak to? (& how often – monitoring)  g. Have family members/carers been involved? If so, how? (if relevant)  h. Overall, how did you feel about recording and monitoring your symptoms? *(prompt about whether they found it reassuring or not)* |
| **INFORMATION RECEIVED ON COVID CARE AT HOME AT ONBOARDING**  4 a. Did you receive information on COVID care at home **in person** from a member of the care team?  If no, move to 4b.  4b. Did you receive information on COVID care at home **over the telephone or a video call** from a member of the care team?  If no, move to 4c.  4c. Were you directed to any information **to read or watch on a website** on COVID care at home?  If no, move to 4d.  4d. Were you directed to any information **to read or watch on an app** on COVID care at home?  If no, move to 5. | a. i) If yes, what information did you receive? (*Monitoring symptoms? Using oximeter? Recording symptoms? Seeking further advice?)*  ii) Who gave you the information?  Was it in your first language?  iii) How easy was it to understand the information?  iv) Did the person you spoke to describe the readings and what they mean in relation to your everyday symptoms and experience?  b. i) If yes, what information did you receive? (*Monitoring symptoms? Using oximeter? Recording symptoms? Seeking further advice?)*  ii) Who gave you the information?  iii) Was it in your first language?  iv) How easy was it to understand the information?  c. i) If yes, were you able to access the information you needed on the website?  ii) If no, why not?  If yes, what information did you receive? (*Monitoring symptoms? Using oximeter? Recording symptoms? Seeking further advice*?)  iii) Was it in your first language?  iv) How easy was it to understand the information?  d. i) If yes, were you able to access the information you needed on the app?  If no, why not?  If i) is yes, what information did you receive? *(Monitoring symptoms? Using oximeter? Recording symptoms? Seeking further advice?)*  iii) Was it in your first language?  iv) How easy was it to understand the information? |
| **CARRYING OUT THE MONITORING**  **OXIMETER**  5. How did you find using the oximeter to monitor your oxygen levels? *(may like to prompt about how often they used it)*  **OTHER SYMPTOMS**  6. Overall, how did you find monitoring your other symptoms (pulse heart rate/temperature/symptoms) at home? *(may like to prompt about how often they monitored other symptoms)* | a. What helped you to use it?  b. What got in the way?  c. What did you do when you had problems? (prompt about type of support and usefulness)  What could be changed to make it easier to use the oximeter?  a. What worked well?  b. What got in the way?  c. What did you do when you had problems? (prompt about type of support and usefulness)  d. What could be changed to make it easier to monitor your other symptoms?  e. Was there any parts of monitoring that you were uncertain about?  f. Did you seek further advice from anyone about monitoring your symptoms? Prompt – who, when, how? |
| **CARRYING OUT RECORDING**  7. Overall, how did you find recording your readings (blood oxygen levels/symptoms/pulse heart rate/temperature)? *(using an app / diary / both) (prompt about how often they recorded their readings)* | a. What worked well?  b. What got in the way?  c. What did you do when you had problems? (prompt about type of support and usefulness)  d. What could be changed to make it easier to monitor your other symptoms?  e. Was there any part of recording your symptoms that you were uncertain about? *(prompt about whether they felt confident)*  r. Did you seek further advice from anyone about recording? Prompt – who, when, how? |
| **COMMUNICATING READINGS TO MEMBER OF THE TEAM**  **8. How have you found sending or communicating your symptoms and readings to the COVID care at home team? (if needed)** | a. What worked well?  b. What got in the way?  c. What did you do when you had problems? (prompt about type of support and usefulness)  d. What could be changed to make it easier to communicate your readings to the COVID care at home team?  e. Was there any part of communicating your readings to a member of the team that you were uncertain about?  r. Did you seek further advice from anyone about communicating your readings? Prompt – who, when, how? |
| **SEEKING FURTHER ADVICE**  8. Have you had to seek further support and help (escalate your care) because of the readings given by your oximeter or because of other things such as a change in symptoms? | a. How did this go?  b. What did this involve?  c. What was your experience of being sent for further support and help (e.g. escalated or admitted to hospital)?  d. What were you instructed to do? (i.e. dial 111, dial 999, go to A&E)?  e. Did you self-escalate your care if necessary? Why or why not? *(prompt on how they made the decision that they needed to seek further help)*  f. What helped you to seek further support?  g. What got in the way of seeking further support?  h. What could be changed to make it easier? |
| **DISCHARGE**  9. What was your understanding about what would happen once you are discharged from COVID care at home? | 1. How did you feel after being discharged from COVID care at home? (prompt about whether they found it reassuring or not?) 2. Since being discharged from COVID care at home, what other services/support have you accessed? *(If discharged from COVID care at home).*    - 1. What were these?      2. How often have you had to access these? |
| **RECOMMENDATIONS**  10. If a friend who was in a similar position to you at the start of your illness was offered COVID care at home, would you recommend it to them  a) over hospital care?  b) over no monitoring, with the option to access usual services as needed.  11. Do you have any recommendations to improve the service? | A) If yes, why? If no, why not?  B) If yes, why? If no, why not?  If yes, what? |
| 12. Is there anything else that you would like to say about what we have talked about? |  |

**If participants did not want to receive COVID care at home**

Could ask Q1-5, and then:

| **Main question** | **Follow up questions (prompts)** |
| --- | --- |
| 1. Please tell me a bit about yourself | - Do you live by yourself or with others? - How long have you lived in your neighbourhood? - Do you have any family or friends living close by? - Is English your first language? If N, ask what is their first language. |
| **FINDING OUT ABOUT THE SERVICE**   1. Can you tell me the story of how you ended up being referred to COVID care at home? | - 1. How were you referred to the service?   2. Who did you speak to and when? (virtual/face to face? How were you involved in the assessment process?) *(for assessment/triage)*   3. When you were first told about the service, how was it described to you?   4. What were your first impressions of COVID care at home? *(prompt about whether they found it reassuring or not)*      1. Positive impressions?   5. Did you have any concerns/worries? |
| **EXPECTATIONS OF COVID CARE AT HOME**   1. Please tell me about what you thought receiving COVID care at home would involve | a. What equipment would you be given?  c. What symptoms would you have had to monitor? For each, ask how often.  d. How would you have had to record your symptoms? (e.g. paper, digital app, telephone line)  e. Were you offered a choice in how you could have recorded your symptoms?  f. Who did you speak to?  h. Overall, how did you feel about the prospect of recording and monitoring your symptoms? *(prompt about whether they found it reassuring or not)* |
| **INFORMATION RECEIVED ON COVID CARE AT HOME AT ONBOARDING**  4 a. Did you receive information on COVID care at home **in person** from a member of the care team?  If no, move to 4b.  4b. Did you receive information on COVID care at home **over the telephone or a video call** from a member of the care team?  If no, move to 4c.  4c. Were you directed to any information **to read or watch on a website** on COVID care at home?  If no, move to 4d.  4d. Were you directed to any information **to read or watch on an app** on COVID care at home?   1. If no, move to 5. | a. i) If yes, what information did you receive? (*Monitoring symptoms? Using oximeter? Recording symptoms? Seeking further advice?)*  ii) Who gave you the information?  Was it in your first language?  iii) How easy was it to understand the information?  iv) Did the person you spoke to describe the readings and what they mean in relation to your everyday symptoms and experience?  b. i) If yes, what information did you receive? (*Monitoring symptoms? Using oximeter? Recording symptoms? Seeking further advice?)*  ii) Who gave you the information?  iii) Was it in your first language?  iv) How easy was it to understand the information?  c. i) If yes, were you able to access the information you needed on the website?  ii) If no, why not?  If yes, what information did you receive? (*Monitoring symptoms? Using oximeter? Recording symptoms? Seeking further advice*?)  iii) Was it in your first language?  iv) How easy was it to understand the information?  d. i) If yes, were you able to access the information you needed on the app?  If no, why not?  If i) is yes, what information did you receive? (Monitoring symptoms? Using oximeter? Recording symptoms? Seeking further advice?)  iii) Was it in your first language?  iv) How easy was it to understand the information? |
|  |  |
| **REASONS FOR DECLINING**   1. Why did you choose not to take part in the CO@h remote monitoring? 2. Could anything be changed to make you want to receive COVID care at home more? | - 1. Did anything get in the way? If so, what?   2. How did this get in the way?   3. If so, what?   4. How would this help? |
| **OTHER CARE/SERVICES ACCESSED**   1. Did you have any other type of monitoring? | - 1. If so, what?   2. How did you find this? |
| 1. Have you had to seek further support and help? (e.g. GP or A&E) | - 1. If so, what? |
| 1. Is there anything else you’d like to say about what we have talked about? | - 1. How would this help? |

**If participants withdrew from receiving COVID care at home**

| **Main question** | **Follow up questions (prompts)** |
| --- | --- |
| 1. Please tell me a bit about yourself | - Do you live by yourself or with others? - How long have you lived in your neighbourhood? - Do you have any family or friends living close by? - Is English your first language? If N, ask what is their first language. |
| **FINDING OUT ABOUT THE SERVICE**   1. Can you tell me the story of how you ended up being referred to COVID care at home? | - 1. How were you referred to the service?   2. Who did you speak to and when? (virtual/face to face? How were you involved in the assessment process?) *(for assessment/triage)*   3. When you were first told about the service, how was it described to you?   4. What were your first impressions of COVID care at home? *(prompt about whether they found it reassuring or not)*      1. Positive impressions?   5. Did you have any concerns/worries? |
| **DESCRIBING COVID CARE AT HOME**   1. COVID care at home is carried out slightly differently in different parts of the country. Please can you tell me about what receiving COVID care at home has involved for you? | a. What equipment were you given?  b. How/when was the pulse oximeter delivered to you? Can you tell me how it works?  c. What symptoms did you have to monitor? For each, ask how often.  d. How did you have to record your symptoms? (e.g. paper, digital app, telephone line)  e. Were you offered a choice in how you recorded your symptoms?  f. Who did you speak to? (& how often – monitoring)  g. Have family members/carers been involved? If so, how? (if relevant)  h. Overall, how did you feel about recording and monitoring your symptoms? *(prompt about whether they found it reassuring or not)* |
| **INFORMATION RECEIVED ON COVID CARE AT HOME AT ONBOARDING**  4 a. Did you receive information on COVID care at home **in person** from a member of the care team?  If no, move to 4b.  4b. Did you receive information on COVID care at home **over the telephone or a video call** from a member of the care team?  If no, move to 4c.  4c. Were you directed to any information **to read or watch on a website** on COVID care at home?  If no, move to 4d.  4d. Were you directed to any information **to read or watch on an app** on COVID care at home?  If no, move to 5. | a. i) If yes, what information did you receive? (*Monitoring symptoms? Using oximeter? Recording symptoms? Seeking further advice?)*  ii) Who gave you the information?  Was it in your first language?  iii) How easy was it to understand the information?  iv) Did the person you spoke to describe the readings and what they mean in relation to your everyday symptoms and experience?  b. i) If yes, what information did you receive? (*Monitoring symptoms? Using oximeter? Recording symptoms? Seeking further advice?)*  ii) Who gave you the information?  iii) Was it in your first language?  iv) How easy was it to understand the information?  c. i) If yes, were you able to access the information you needed on the website?  ii) If no, why not?  If yes, what information did you receive? (*Monitoring symptoms? Using oximeter? Recording symptoms? Seeking further advice*?)  iii) Was it in your first language?  iv) How easy was it to understand the information?  d. i) If yes, were you able to access the information you needed on the app?  If no, why not?  If i) is yes, what information did you receive? (Monitoring symptoms? Using oximeter? Recording symptoms? Seeking further advice?)  iii) Was it in your first language?  iv) How easy was it to understand the information? |
| **REASON FOR WITHDRAWING FROM CO@H**   - - 1. Why did you choose to withdraw from receiving COVID care at home? | - 1. - Did anything get in the way? If so, what?   - How did this get in the way? |
| **CARRYING OUT THE MONITORING (if applicable)**  **OXIMETER**   - - 1. How did you find using the oximeter to monitor your oxygen levels?   **OTHER SYMPTOMS**   - - 1. Overall, how did you find monitoring your other symptoms (pulse heart rate/temperature/symptoms) at home? | a. What helped you to use it?  b. What worked less well?  c. What did you do when you had problems? (prompt about type of support and usefulness)  What could be changed to make it easier to use the oximeter?  a. What worked well?  b. What worked less well  c. What did you do when you had problems? (prompt about type of support and usefulness)  d. What could be changed to make it easier to monitor your other symptoms? |
| **CARRYING OUT RECORDING (If applicable)**   - - 1. Overall, how did you find recording your readings (blood oxygen levels/symptoms/pulse heart rate/temperature)? *(using an app / diary / both)* | a. What worked well?  b. What worked less well?  c. What did you do when you had problems? (prompt about type of support and usefulness)  d. What could be changed to make it easier to monitor your other symptoms?  e. Was there any part of recording your symptoms that you were uncertain about?  r. Did you seek further advice from anyone about recording? Prompt – who, when, how? |
| **COMMUNICATING READINGS TO MEMBER OF THE TEAM (If applicable)**  9. How have you found sending or communicating your symptoms and readings to the person taking the readings (if needed) | a. What worked well?  b. What worked less well?  c. What did you do when you had problems? (prompt about type of support and usefulness)  d. What could be changed to make it easier to communicate your readings to a member of the team?  e. Was there any part of communicating your readings to a member of the team that you were uncertain about?  f. Did you seek further advice from anyone about communicating your readings? Prompt – who, when, how? |
| **SEEKING FURTHER ADVICE (If applicable)**   - - 1. Have you had to seek further support and help (escalate your care) because of the readings given by your oximeter or because of other things such as a change in symptoms? | 1. How did this go? 2. What did this involve? 3. What was your experience of being sent for further support and help (e.g. escalated or admitted to hospital)? 4. What were you instructed to do? (i.e. dial 111, dial 999, go to A&E)? 5. Did you self-escalate your care if necessary? Why or why not? 6. What helped you to seek further support? 7. What got in the way of seeking further support? 8. What could be changed to make it easier? |
| **OTHER CARE/SERVICES ACCESSED**  10. Did you have any other type of monitoring? | - 1. - If so, what?   2. -How did you find this? |
| 11. Have you had to seek further support and help? (e.g. GP or A&E) | - 1. - If so, what? |
| **RECOMMENDATIONS**  12. Could anything be changed to make you want to receive COVID care at home more?  13. If a friend who was in a similar position to you at the start of your illness was offered COVID care at home, would you recommend it to them  a) over hospital care?  b) over no monitoring, with the option to access usual services as needed.  14. Do you have any recommendations to improve the service? | 1. If so, what? 2. How would this help?   A) If yes, why? If no, why not?  B) If yes, why? If no, why not?  If yes, what? |
| 15. Is there anything else you’d like to say about what we have talked about? |  |

**Interview questions on demographic characteristics (asked at the end of the interview)**

- Patient or carer? (relationship with patient, if carer)
- Gender
- Age
- How many people do you live/cohabit with?
- Which of these best describes your living arrangement? *Please select one answer (I own my home outright, I own my home with a mortgage, I rent from local authority/housing association, I rent privately, Other (e.g. living with family/friends), prefer not to say)*
- Ethnicity of patient (and family member if applicable)
- At what age did you complete your continuous full time education? *(__ years/never went to school, do not wish to answer)*
- Which of these best describes your highest *educational* qualification? *(Please select one answer) (No formal qualification, GCSE/CSE/O level or equivalent, A level/AS level or equivalent, Degree level or higher, Other (please specify), do not wish to answer)*
- Which of these best describes your current work situation? *(please tick all that apply) (Working full time, working part time, self-employed, student in higher education, unemployed, homemaker, retired, furloughed under COVID-19, Full time carer (of dependent child or adult), not in work due to poor health or disability,, Other (if other please describe), do not wish to answer).*
- Is English your first language? *(yes/no/do not wish to answer)*
- Prior to your current illness, are your day-to-day activities limited because of a health problem or disability which has lasted, or is expected to last, at least 12 months?? (Includes problems which are due to old age.) (yes, limited a lot/yes, limited a little, no/do not wish to answer)
- What is your Postcode? (optional)
