# Supplementary material for: Patients' experiences of, and engagement with, remote home monitoring services for COVID‐19 patients: A rapid mixed‐methods study
Source: Health Expect. 2022 Jul 7;25(5):2386–404. doi: 10.1111/hex.13548 (PMC9349790; doi:10.1111/hex.13548)
Supplement: Supplementary file 3 — Supporting information. [file HEX-25--s003.docx]

*Appendix 3.* Patient/carer recommendations to improve the service

| **Category** | **Recommendation** |
| --- | --- |
| Patient awareness | - Need more publicity about the service - Need to know about the service sooner or earlier referral - Need to improve link to NHS Track and Trace - Need national standardised approach or automatic referral |
| Patient enablement | - Need more information provision about the service at referral, about escalation and about discharge - Need a dedicated contact number for patients to contact service if needed - Some patients require some face-to-face visits - Need reminders to submit readings |
| Workforce | - More continuity of staff and continuity of information - More contact with doctors desired by some patients |
| Individual differences | - More personalised approach - Flexibility of methods and patient choice |
| Equipment | - Provision of thermometers - Support with using technology (oximeter and digital platforms) |
| Logistics of service | - Consider timing of monitoring calls and having a specific time slot - Need to receive calls when promised - Need to ensure practical and efficient arrangement of oximeter delivery and return |
| Signposting | - Following patients up after discharge - A point of contact after discharge to ask questions to - Signposting to places that can support them whilst on the service - Community drives to ease concerns over hospitals |
